# The Tetraspanin CD151 in Papillomavirus Infection

**DOI:** 10.3390/v6020893

**Published:** 2014-02-18

**Authors:** Konstanze D. Scheffer, Fedor Berditchevski, Luise Florin

**Affiliations:** 1 Department of Medical Microbiology and Hygiene, University Medical Centre of the Johannes Gutenberg University, Obere Zahlbacher Strasse 67, 55131 Mainz, Germany; E-Mail: scheffek@uni-mainz.de; 2 School of Cancer Sciences, The University of Birmingham, Birmingham B15 2TT, UK; E-Mail: f.berditchevski@bham.ac.uk

**Keywords:** papillomavirus, HPV, tetraspanin, CD151, receptor, virus entry, L1, L2, endocytosis, trafficking

## Abstract

Human papillomaviruses (HPV) are non-enveloped DNA tumor viruses that infect skin and mucosa. The most oncogenic subtype, HPV16, causes various types of cancer, including cervical, anal, and head and neck cancers. During the multistep process of infection, numerous host proteins are required for the delivery of virus genetic information into the nucleus of target cells. Over the last two decades, many host-cell proteins such as heparan sulfate proteoglycans, integrins, growth factor receptors, actin and the tetraspanin CD151 have been described to be involved in the process of infectious entry of HPV16. Tetraspanins have the ability to organize membrane microdomains and to directly influence the function of associated molecules, including binding of receptors to their ligands, receptor oligomerization and signal transduction. Here, we summarize the current knowledge on CD151, and CD151-associated partners during HPV infection and discuss the underlying mechanisms.

## 1. Introduction

Human Papillomaviruses belong to the family of *Papillomaviridae*, a widespread virus family that infects almost all mammalian species and birds. Based on DNA sequence analyses, over 200 different subtypes have been identified so far, of which there are 170 which are known to infect humans [1,2]. Depending on the HPV subtype, the productive infection can cause the formation of benign warts or malignant tumors, such as cervical cancer. In fact, 99.8% of all cervical cancer cases are attributed to a persistent infection with a so-called high-risk HPV type. The high-risk types HPV16 and HPV18 are alone responsible for over 70% of cervical cancer cases [3]. Human papillomaviruses are small non‑enveloped viruses with a circular double stranded DNA genome, 8 kb in size. The genome includes a non-coding region (LCR = long control region) and, depending on the subtype, eight or nine open reading frames. The DNA-region has binding sites for different cellular transcription factors and regulates the expression of the viral early and late genes. The early genes E1, E2, E4, E5, E6 and E7 are non-structural viral proteins responsible for viral replication and transcription, as well as for transformation, segregation and tumorigenesis [1,4,5]. The approximately 55 nm diameter in size HPV capsid is composed of the two structural proteins: the major capsid protein L1 and the minor capsid protein L2. The capsid contains 360 copies of L1 molecules and a so far unknown number of L2‑copies: 12, 32 or up to 72 per capsid have been identified up until now [6,7]. The capsid proteins L1 and L2 are key players in early events of infection, such as virus binding at the plasma membrane, entry into the cell, and transport of the viral DNA into the nucleus [6,8,9]. So-called pseudovirions (PsVs) are widely used to analyze HPV biology and mechanisms of infection. These PsVs are composed of a viral pseudogenome that encodes a reporter gene encapsidated by the capsid proteins, L1 and L2. These virions are generated by cotransfection of codon optimized L1 and L2 genes together with a reporter plasmid which encodes luciferase or GFP [6,10]. Reporter-gene expression after PsVs‑infection of target cells is used as readout for successful cell entry after delivery of the viral pseudogenome to the nucleus.

The infectivity cycle of HPV is a multistep process and relies on the interplay of viral proteins with a wide range of cellular cofactors [8]. For HPV16, it was shown that the first step involves binding of the viral capsid to heparan sulfate proteoglycans (HSPGs) such as syndecan-1 or to a non-HSPG component of the extracellular matrix (ECM) such as laminin-332 [11,12]. After conformational changes in both capsid proteins, the virus is transferred to a secondary receptor complex [13]. There is increasing evidence that early events of post-viral binding and transfer of virions to the secondary receptor complex involves tetraspanin proteins and tetraspanin enriched microdomains (TEMs or TERMs) [8,14,15,16]. After cellular entry at CD151- and CD63-positive TERMs, virus capsids are accumulated in CD63 positive intracellular endocytic compartments, where capsid disassembly and uncoating of the viral DNA occurs [14,17]. Subsequently, the minor capsid protein L2 chaperones the viral DNA into the host cell nucleus to subnuclear structures known as promyelocytic leukemia nuclear bodies (PML-NBs) or nuclear domain 10 (ND10), which represent the sites of viral transcription and replication [8,9,18,19].

With CD151 playing important roles in various aspects of integrin-dependent cellular responses, its involvement during the early stages of HPV infection is likely to go beyond a simple lateral capsid‑tethering function [15,16]. The current knowledge on the involvement of CD151 and associated factors in the HPV life cycle is summarized in this review.

## 2. The Tetraspanin CD151

**Figure 1:**
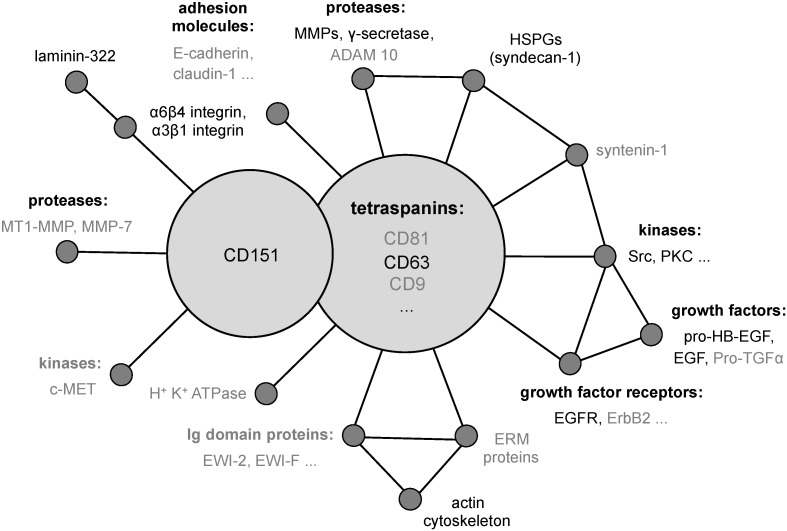
Schematic diagram showing published tetraspanin partners. Proteins essential for HPV16 infection are shown in black [22,23,24,28,29,30,31,32,33,36,37,38,39,40,41,42].

The tetraspanin CD151/TSPAN 24/PETA3 was originally identified in platelet and endothelial cells by using a monoclonal antibody against human acute myeloid leukaemia cells [20]. It was cloned as SFA-1 from leukemia cell lines [21] and has since been found in many different cell types [22,23,24]. In addition, CD151 is frequently overexpressed on cancer cells and is functionally linked to cancer metastasis [25]. Like all members of the tetraspanin family, CD151 consists of four transmembrane domains, two extracellular loops (EC1 and EC2), a short intracellular loop and NH2- and COOH‑terminal cytoplasmic extensions [26,27]. Through its ability to associate with a variety of proteins including other tetraspanin proteins (Figure 1), CD151 may control functional interplay between TERM-associated receptors and enzymes such as laminin-binding integrins (α3β1, α6β4), growth factor receptors (EGFR, ErbB2, c-Met), and proteases (MMPs, ADAM proteins) [28,29,30,31,32,33]. This lateral coordination of signaling pathways and enzymatic activities of the proteins is likely to underlie the modulatory activity of CD151 in cell adhesion, migration and proliferation [32,34,35].

## 3. The Early Events of HPV Infection and the Functional Role of CD151

### 3.1. Primary Binding and Interaction Partners on the Cell Surface

**Figure 2:**
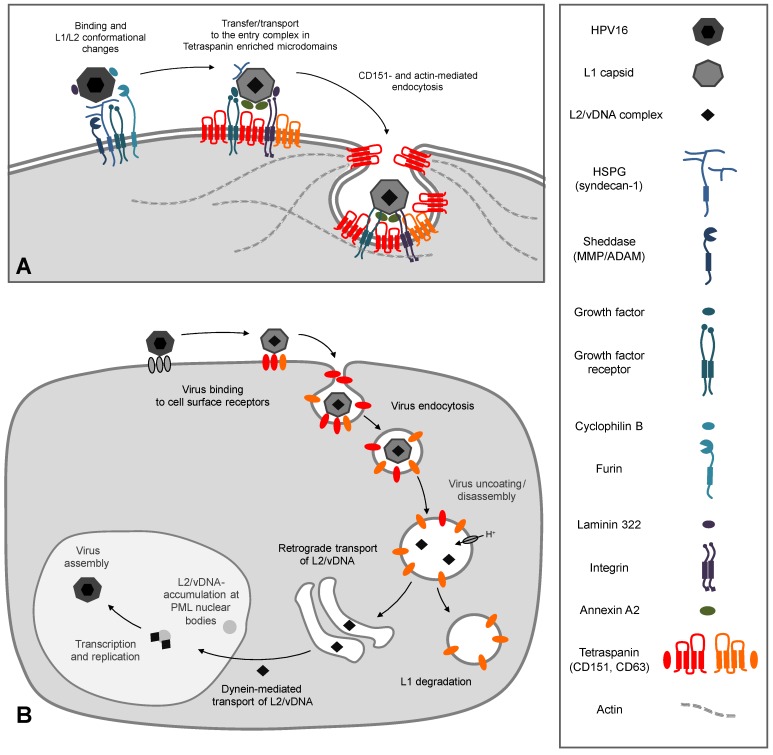
HPV16 infection: (**A**) Cell surface events. Virus particles bind to heparan sulfate proteoglycans (HSPGs) and non-HSPG proteins (laminin-332, growth factors) inducing activation of growth factor receptors and integrins. After conformational changes of both capsid proteins by HSPG binding, cyclophilin B, and furin, papillomavirus particles become transferred to the second receptor complex (CD151, CD63, annexin A2, laminin, α6-integrin, growth factors, and their receptors) in tetraspanin enriched microdomains. The tetraspanin CD151 and actin mediate a clathrin-, caveolin-, and dynamin-independent endocytosis of the virus probably by membrane budding and vesicle scission. (**B**) Virus binding and uptake leads to virus accumulation in CD63 positive endosomes. Acidification and cyclophilin B enable capsid disassembly and uncoating of the infectious complex (L2/vDNA). L2-mediated trafficking leads to accumulation of the L2/vDNA complex at PML nuclear bodies. The differentiation program of infected keratinocytes controls virus transcription and replication. Finally, new HPV particles are assembled in the host-cell nucleus.

HPV gain access through micro lesions of skin or mucosa to the basal dividing cells of the epithelium [43]. Primary binding partners of most HPV subtypes are heparan sulfate proteoglycans (HSPGs) [44,45,46,47,48]. These are highly glycosylated proteins that are either associated with the cell surface (syndecan-1 and glypicans) or secreted and subsequently incorporated into the basement membrane (perlecan) [49,50]. The primary association with the negatively charged HSPGs is mediated by positively charged specific lysine residues of the major capsid protein L1 [46,48]. This interaction causes conformational changes within the capsid which leads to lower affinity for HSPGs allowing transfer of viral particles to a secondary receptor [51,52,53,54,55,56]. Further conformational changes of the capsid mediated by the peptidyl-prolyl cis/trans isomerase cyclophilin B [54], cleavage of the exposed L2-N-terminus by the pro-protein convertase furin [57,58], and the association with additional proteins such as laminin-322 [59,60], growth factors (e.g., EGF, FGF) [61] and annexin [62,63] is likely to play a key role in HPV-interaction with a secondary receptor, and necessary for productive infection (Figure 2A).

### 3.2. The Secondary Receptor Complex

The true identity of a secondary receptor, which is responsible for the internalization of HPV, is still hotly debated in the research community. One of the putative candidates is the α6 integrin complex (α3β1, α6β4) [15,64,65,66,67,68]. This complex is able to bind the HPV L1 protein which leads to integrin-dependent activation of the focal adhesion kinase (FAK) and the phosphoinositide 3-kinase (PI3K) required for infectious entry of HPV [68,69,70,71]. The epidermal growth factor receptor (EGFR) is another putative secondary target for binding and cellular entry of viral particles. In fact, it was shown that HPV16 binds to complexes composed of HSPGs and growth factors (GF) [61]. Mechanistically, it has been proposed that after constitutive HSPG-shedding by matrix metalloproteases (MMPs), HSPG/GF-decorated HPV16 complexes are transferred to EGFR and activate the PI3K/Akt/mTOR pathway. HSPG-shedding could provide an alternative mode of virus transfer to a secondary receptor. In addition, early activation of signaling cascades by binding of HPV16 to target cells may contribute to the formation of “HPV entry platforms” (HPEP—Human Papillomaviruses Entry Platforms) as a precondition for viral entry. Interestingly, α6 integrin complexes and EGFR are associated with tetraspanin microdomains [33,72], thus suggesting that tetraspanins may control the assembly and/or spatial organization of secondary receptors within the HPEP (Figure 2A).

In addition to L1-based interactions with primary and secondary receptors, virions also utilize the minor capsid protein L2. It has been shown that L2 specifically interacts with the S100A10 subunit of the annexin A2 heterotetramer on the surface of keratinocytes which contributes to HPV16 internalization and infection of epithelial cells [62,63]. HPV16-induced activation of the EGFR initiates an increased annexin A2 translocation to the plasma membrane [63] and precipitation studies suggest that HPV16, EGFR and annexin A2 could form a functional complex. Additionally, inhibition with a specific antibody and siRNA/shRNA-mediated depletion of annexin A2 and SP100A10 reduced HPV16 internalization [62,63]. Due to interaction of annexin A2 with TERM-associated proteins, it is tempting to speculate that the recruitment of annexin A2 to TERMs serves to further facilitate compartmentalization of viral particles in HPEP and potentiate viral entry.

### 3.3. CD151 and its Functional Role during Early Events of Infection

HPV infect only the basal epithelial cells of the skin and mucosa, and both EGFR and α6 integrins are highly expressed in the basal layer of epithelium. Importantly, where tested CD151 was the only tetraspanin which mimics the tissue distribution pattern of EGFR and α6 integrin. This suggests that a complex of CD151 with laminin-binding integrins (and, possibly, with EGFR) can be formed *in vivo* and this may be critical for post-binding transfer of HPV to HPEP [15,24]. Indeed, it has been demonstrated, that siRNA mediated depletion of CD151, α3β1 and α6 integrin complexes in different cell lines reduced the infection of HPV16 [15].

An additional indication for the involvement of CD151 in HPV16 infection was provided by colocalization studies using confocal microscopy. It has been observed that only a few viral particles were colocalized with CD151 at the early time points of HeLa cells infection (up to 10 minutes), but as the infection process proceeded further, colocalization of CD151 and HPV16-L1 was increased. The association of viral particles with CD151 was also evident in intracellular compartments seven hours after infection [14]. Similarly, colocalization of viral particles with CD151 was observed in cytoplasmic vesicular structures of infected primary keratinocytes [15]. Colocalization of CD151 and HPV was confirmed by electron microscopy where immunogold-labeled CD151 and HPV were found on the plasma membrane and during membrane invagination [8,14]. Interestingly, total internal reflection fluorescence (TIRF) microscopy of live cells showed the lateral movement of colocalized viral particles and CD151. Importantly, only colocalized CD151 and HPV16 were endocytosed, while CD151-free viral particles remained on the plasma membrane [15]. These recently published data suggest that the association of viral particles with CD151 at the plasma membrane is a prerequisite for viral internalization. Furthermore, these results point to an important role of CD151 as a vehicle for delivery of cell-bound HPV to their entry platforms (HPEP). Whilst the molecular mechanisms underlying CD151-dependent lateral movements of HPV remain completely unknown, the involvement of TERM-associated receptors in early events of HPV16 infection can certainly lend speculation regarding the contribution of integrin- and EGFR-triggered signaling pathways. It is also conceivable that CD151 is involved in the transferring of HPV from the primary to secondary receptor complexes. In this regard, it has been recently demonstrated that the interaction between the cytoplasmic domains of syndecan-1 and integrin leads to the assembly of a bigger protein complex on the plasma membrane of epithelial cells. This complex also includes ErbB2 and laminin-332, and the complex formation subsequently activates the PI3K/Akt/mTOR pathway [73]. ErbB2 belongs to the family of growth factor receptors and is the preferred dimerization partner of the other growth factor receptors (EGFR, ErbB3 and ErbB4) [74,75]. Although the role of CD151 in the assembly of the syndecan-1/α6β4/ErbB2/laminin-332 complex is yet to be established, recently published data showed that CD151 might influence molecular interactions involving ErbB-receptors [76]. These multiple requirements for infectious internalization of papillomaviruses could be the reason for the observed extremely slow internalization kinetics of viral particles (with half times of internalization up to 14 h) compared to other viruses [77,78,79]. About 2 h were needed for the first viruses to be internalized [70] which correlates with the association kinetics of viral particles with the tetraspanins CD151 and CD63 on the cell surface [14] and personal communication [80].

### 3.4. Function of CD151 during HPV Endocytosis

**Figure 3:**
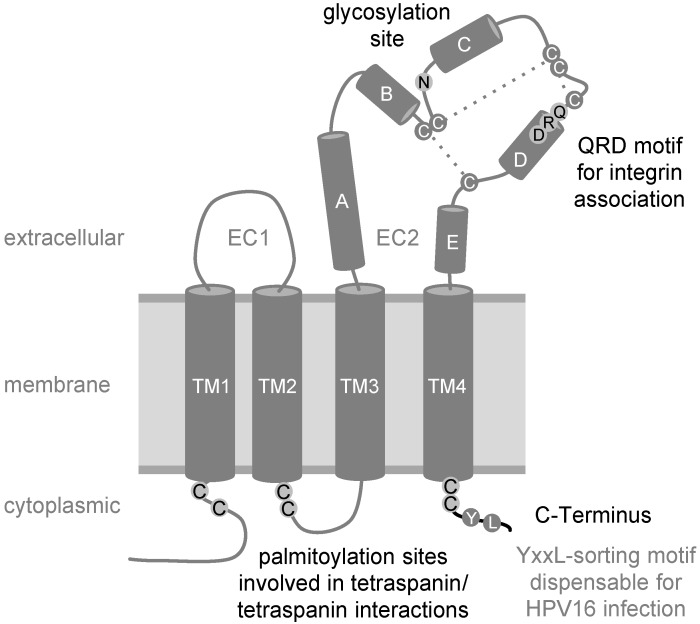
Schematic view of tetraspanin CD151 with four transmembrane domains (TM1‑4), two extracellular domains (EC1, EC2) with helices A–E, and three disulfide bonds (dashed lines). Some amino acids were represented in one-letter code. Residues important for HPV16 endocytosis are shown in black: The juxtamembrane cysteines that constitute potential palmitoylation sites, the N-glycosylation site, the QRD-motif required for integrin interaction and the C-terminus of the CD151 molecule.

Currently, it seems to be widely accepted that different HPV subtypes share similar endocytic strategies for cell entry. The exclusion of key players of well-characterized pathways by means of multiple methodological approaches has led to the notion that HPV uses a novel clathrin-, caveolin-, and dynamin-independent endocytosis pathway for infection [14,16,70]. These studies also demonstrated that virus internalization requires tyrosine kinase signaling, actin dynamics, and function of CD151 [15,16,70]. Specifically, depletion of CD151 in HeLa cells led to decreased endocytosis of viral particles and reduced disassembly of viral capsids in endocytic compartments [15]. Expression recovery experiments using well-characterized CD151 mutants provided an important insight into underlying molecular mechanisms (summarized in Figure 3). The CD151 C-terminus contains a Y^245^-RSL-sequence, representing an YXXФ endocytosis/sorting motif [81]. The YXXФ sorting motif in the cytoplasmic domain of transmembrane proteins is recognized by the adaptor protein complexes (AP adaptors), which link cargo proteins to clathrin-dependent endocytic trafficking [82], and it is predicted that two point mutations within the CD151 sorting motif (YRSL → ARSA, CD151-Yala mutant) would lose their ability to interact with AP complexes. Accordingly, not only was antibody-induced internalization of CD151-Yala inhibited, but overexpression of this mutant also suppressed endocytosis of the associated integrins [81]. Surprisingly, CD151-Yala could restore endocytosis and capsid disassembly of HPV16 in expression recovery experiments using HeLa cells [15]. These data showed that CD151 does not function as a mere vehicle for HPV16 uptake and confirms further that HPV enter the target cells using clathrin-independent mechanisms [14,16]. In contrast, expression recovery experiments also demonstrated that the association with integrins is critical for the activity of CD151 towards HPV. Indeed, a CD151-mutant which lost the ability to interact with integrins after mutation of the glutamine-arginine-aspartic acid motif (CD151-QRD) [27,83], could not restore virus endocytosis and capsid disassembly. Furthermore, deletion of the C-terminal cytoplasmic region (CD151-∆C) also causes CD151 to lose its activity towards HPV16. Palmitoylation of CD151 seems to be equally important for endocytic entry and post-endocytic processing of HPV. While the CD151 palmitoylation-deficient mutant retains the ability to interact with integrins, it fails to recruit the complexes to TERMs [84]. Thus, other components of TERMs in target cells must contribute to the post-binding steps in the HPV life cycle. Whilst EGFR and MMPs are the obvious candidates to fulfill this role (see above), our recent results indicate the involvement of various other TERM-associated proteins and complementary pathways (unpublished work [85]). Furthermore, our results with the CD151‑∆C mutant, which can associate with integrins and recruit the complexes to TERMs, suggest that the presence of CD151 in TERMs and the C-terminal cytoplasmic portion of the protein seem to be critical for activation of these pathways.

One of the possible targets for CD151-dependent TERM-associated pathways is the actin cytoskeleton. It has been reported that actin polymerization is a precondition for viral entry into the cell [70,86]. Inhibition of actin polymerization resulted in the formation of long tubules, which were unable to separate from the plasma membrane [70]. The authors proposed that actin polymerization is required for vesicle scission and, therefore, for successful endocytosis of viral particles. In this regard, CD151 is known to regulate actin cytoskeleton dynamics via small Rho GTPases [87,88,89]. Furthermore, as shown for HPV16 internalization, endocytosis of CD151 depends on phosphatidylinositol 3-kinase (PI3K) activity [81] supporting the connection between signaling pathways, tetraspanins and actin as preconditions for HPV16 internalization. In addition to CD151, other tetraspanins in TERMs may be functionally linked to the actin cytoskeleton. For example, CD9 and CD81 are associated with members of the immunoglobulin superfamily, EWI-2 and EWI-F [36,37,38]. Both EWI-2 and EWI-F were found to interact with ERM-proteins (ezrin, radixin and moesin), which function as key regulators of actin-dependent dynamics of the plasma membrane [39,40]. In addition, EWI-2 associates with the actin-binding protein α-actinin [41] and the C-terminal cytoplasmic domain of CD81 is also able to bind directly to ERM-proteins [40], which supports the notion that TERMs contribute to actin cytoskeleton reorganization during viral cell entry. Furthermore, ERM-proteins and the EWI-2-α-actinin complex association are linked to the infection process of other pathogens, such as HIV: while moesin supports HIV membrane fusion [42], EWI-2-α-actinin negatively regulates infection [41]. Thus, it is conceivable that all these tetraspanin-driven interactions may contribute to receptor clustering, budding and scission of virus-filled vesicles thereby inducing virus internalization into the cell. Further characterization of protein interaction dynamics within TERMs will be necessary to unravel intricate functional connections between various TERM components, which control early steps of the HPV infection.

## 4. Intracellular Trafficking of Capsid Proteins

After association and cointernalization of viral particles with the tetraspanins CD151 and CD63 [14,15], viruses are trafficked towards perinuclear CD63 containing vesicles (Figure 2B). CD63 is predominantly localized in intracellular vesicles such as late endosomes and lysosomes [90]. Acidification of these endocytic vesicles mediated by the vacuolar ATPase ([91] and unpublished work [92]) enables HPV capsid disassembly and release of the L2/genome complex [70,77,93]. Cellular chaperones such as cyclophilin B facilitate the dissociation of the L1 protein from the L2/DNA complex [94] leading to separation of L1 and L2 into different compartments. L2 uses the retrograde pathway for chaperoning the viral genome to the Golgi compartment [91,95]. The minor capsid protein is now able to interact with a variety of cellular proteins essential for trafficking and crossing of intracellular membranes (for review see [8,9]). After leaving intracellular compartments, the L2/DNA-complex binds to dynein, a microtubule motor protein-complex, and is transported along the microtubule network towards the host-cell nucleus [96,97]. Ultimately, the L2-mediated transport leads to accumulation of the L2/DNA-complex at subnuclear structures, the PML nuclear bodies [19,98,99]. PML-accumulation is required for manifestation of infection, stimulation of cell proliferation, vegetative amplification of the genome, and, finally, papillomavirus morphogenesis [8,100,101,102,103,104].

## 5. Conclusions and Future Directions

Although it was shown that entry of HPV16, 18 and 31 is mediated by the tetraspanin CD151 in a clathrin-, caveolin- and dynamin-independent manner, there is no evidence that HPV interact with CD151 directly. Thus, it is likely that the main function of CD151 during the initial steps of HPV infection is to coordinate the sequential passage of viral particles between many HPV-binding surface proteins, (*i.e.*, HSPGs, laminin-binding integrins, growth factors and their receptors) and assembly of HPEP—human papillomaviruses cell entry platforms. Since it was shown that repression of annexin A2/S100A10 interaction, depletion of CD151, and depolymerization of the actin cytoskeleton are able to inhibit HPV endocytosis, it seems plausible that HPEP-associated receptors function to control actin reorganisation and induce membrane invagination and vesicle scission. Whilst receptor-mediated signaling has an essential role in these processes, the involvement of CD151 and other tetraspanins as required modulators remain to be determined. Thus, further work is now required to unravel the contribution of individual tetraspanins in the dynamics of virus-receptor interactions outside and within HPEP. Quantitative proteomics of virus/receptor-complexes on the plasma membrane or in virus-containing endosomes (as described in [105]) will uncover precise molecular composition and stoichiometry of these membrane platforms. In addition, advanced imaging approaches such as super resolution or correlation microscopy will be helpful to examine viral dynamics on the cell surface in more detail. Functional links between HPEP-associated receptor with the actin cytoskeleton also needs further clarification. Tetraspanins are able to interact with actin-linker proteins (ezrin, radixin, moesin; ERM, α-actinin) directly or via binding-partners such as EWI-proteins. These interactions should be investigated in the context of both initial virus-receptor mediated signaling and during the subsequent endocytic steps.

Furthermore, intracellular trafficking of viral particles resulting in capsid disassembly and uncoating of the L2/vDNA complex is still poorly understood. Co-localisation of endocytosed HPV with tetraspanins (e.g., CD151 and CD63) or tetraspanin partners seem to suggest further involvement of TERM-associated proteins in transport, sorting, budding, or fusion of virus-filled endosomes. Depletion of tetraspanins or tetraspanin-regulating proteins in combination with electron- and super resolution microscopy and other advanced imaging strategies should provide important mechanistic insights into virus-utilized or -induced tetraspanin functions in endocytosis and vesicle-trafficking.
